# Influence of Hollow Glass Microspheres (HGM) on Properties of Alkali-Activated Slag Lightweight High-Strength Concrete Under Varying Lightweight Aggregate (LWA) Dosages

**DOI:** 10.3390/ma18143233

**Published:** 2025-07-09

**Authors:** Liankun Wang, Zefeng Lu, Long Cheng, Jun Sun, Yao Huang, Xin Cheng, Minrong Wang

**Affiliations:** School of Civil Engineering and Architecture, Wuyi University, Jiangmen 529020, China; fengzheng403@126.com (L.W.); 2112213012@wyu.edu.cn (Z.L.); 2112213008@wyu.edu.cn (L.C.); s504413396@163.com (J.S.); 15180216366@163.com (Y.H.); 15079261037@163.com (X.C.)

**Keywords:** hollow glass microspheres, lightweight aggregate, alkali-activated slag, lightweight high-strength concrete, microstructure

## Abstract

As a promising and sustainable construction material, alkali-activated slag lightweight high-strength concrete (AAS-LWHSC) may be influenced by lightweight aggregate (LWA) content. In this study, the effects of hollow glass microspheres (HGM) replacing granulated ground blast furnace slag (GGBFS) under varying LWA dosages on the workability, dry apparent density, mechanical properties, and microstructure of AAS-LWHSC were investigated. The results indicated that the dry density of concrete was significantly reduced by HGM, while the “ball-bearing” effect of HGM was observed to enhance workability at a dosage of 6%. The 7-day mechanical properties of AAS-LWHSC were found to decline progressively with increasing HGM content. However, at the shale ceramsite sand replacement rates of 35% and 65%, the incorporation of 6% HGM slightly improved the 28-day mechanical properties. Due to the absence of the water-releasing effect from shale ceramsite, the pozzolanic reactions of HGM were restricted, resulting in coarse hydration products and a reduction in the mechanical performance of AAS-LWHSC.

## 1. Introduction

Lightweight high-strength concrete (LWHSC), which exhibits lower self-weight and broader application potential compared to conventional concrete [1], is generally defined as having a density below 2000 kg/m^3^ and a compressive strength exceeding 40 MPa [2,3,4,5]. LWAs are considered essential components for LWHSC production, with materials such as pumice [6], shale ceramsite [7], perlite [8], expanded clay particles [9], and expanded polystyrene beads [10] being incorporated to reduce concrete density. Despite their low density, LWAs are porous materials [1], and their volumetric content has been demonstrated to significantly influence concrete performance [11,12]. To mitigate LWA-related drawbacks, a method proposed by Shi involves the crushing of large-sized LWAs into smaller particles to enhance mechanical properties [13]. LWHSC with a compressive strength exceeding 100 MPa was developed by Pan using pottery sand (PS) and HGM [14], while Wang achieved an LWHSC with a dry apparent density of 1950 kg/m^3^ and a compressive strength of 120 MPa through the incorporation of PS and steel fibers [15]. In addition to small-sized LWAs, HGM has been widely adopted as a high-performance material for LWHSC development.

HGM, appearing as a white powder macroscopically, is characterized microscopically as hollow spherical particles with thin walls. Its low thermal conductivity, reduced density, high strength, and corrosion resistance [16] make it a critical filler for constructing lightweight multifunctional composites [17]. The optimal compressive and flexural strength improvements were reported by Aslani [18] in concrete containing high-strength HGM, a finding corroborated by Chen [19], who noted that high-strength HGM exhibits a slower decline in compressive strength, smaller average particle size, and more uniform distribution within the concrete matrix. The SiO_2_-rich composition of HGM enables pozzolanic reactions, which have been shown to enhance interfacial bonding with cementitious matrices [20,21]. Numerous studies have utilized HGM for LWHSC development. For instance, ultra-high-performance concrete (UHPC) with a compressive strength of 160 MPa and a dry density of 1862 kg/m^3^ was fabricated by Dahal [22] through high-temperature curing with substantial HGM incorporation. Similarly, LWHSC with compressive strengths above 120 MPa and dry apparent densities reduced to 1800 kg/m^3^ was developed by Lu [23] by replacing 0%, 10%, 30%, and 50% of cement volume with HGM.

With the ongoing transition toward green and low-carbon engineering materials, alkali-activated binders (AAB) composed of industrial byproducts such as GGBFS, fly ash (FA), and silica fume (SF) have increasingly replaced conventional cement [24]. AAB systems predominantly based on GGBFS, termed alkali-activated slag (AAS) [25], primarily yield aluminum-containing C-S-H gels as reaction products due to the high silicon, calcium, and aluminum content of GGBFS [26,27]. In an AAS system, LWHSC with a density below 1500 kg/m^3^ and a compressive strength exceeding 70 MPa was developed by Chen [28] using HGM and fly ash cenospheres as primary aggregates. AAS is characterized by significant strength, low permeability, and high resistance to sulfate and acid attack [29,30]. Compared to OPC concrete, greenhouse gas emissions can be reduced by over 40%. Additionally, LWHSC preparation predominantly employs LWAs, resulting in reduced aggregate consumption and lower construction costs for equivalent volumes when contrasted with conventional concrete [31]. Environmental and economic benefits of AAS-LWHSC incorporating 100% lightweight aggregates were further validated by Zheng [32].

However, in most existing studies, the excellent physical properties of HGMs have been utilized primarily for developing various types of concrete. The effects of HGMs on the fundamental mechanical properties of AAS-LWHSC prepared by incorporating HGMs while adjusting the proportion of large and small LWAs under different LWA dosages have not been systematically investigated. Three replacement ratios of shale ceramsite sand (35%, 65%, and 100%) were adopted to substitute shale ceramsite, while the effects of four HGM dosages (3%, 6%, 9%, and 12%) replacing GGBFS on mechanical performance were evaluated. Microstructural analyses were concurrently conducted to elucidate the underlying mechanisms.

## 2. Materials and Methods

### 2.1. Materials

**Figure 1:**
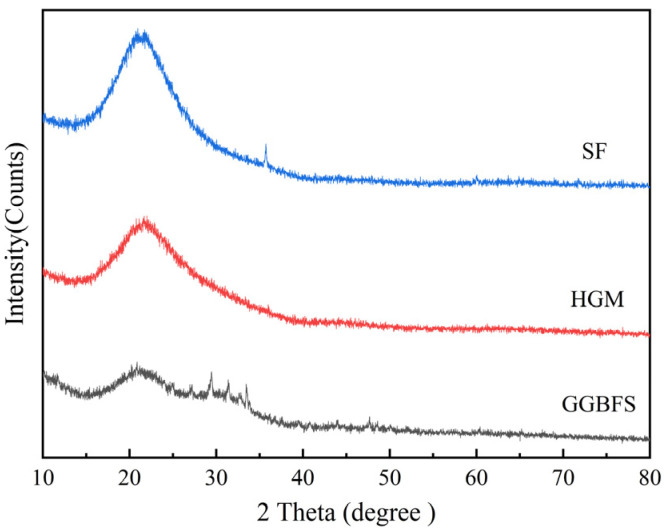
XRD patterns of GGBFS, HGM, and SF.

GGBFS, SF, and HGM were provided by Henan Jinrun New Materials Company (Zhengzhou, China), with their chemical compositions detailed in Table 1. GGBFS was primarily composed of CaO, SiO_2_, and Al_2_O_3_, accompanied by minor MgO. HGM and SF were predominantly characterized by SiO_2_ content. The X-ray diffraction (XRD) patterns of raw GGBFS, SF, and HGM are presented in Figure 1. It can be observed that HGM exhibited an amorphous structure, indicating potential pozzolanic reactivity [33].

**Figure 2:**
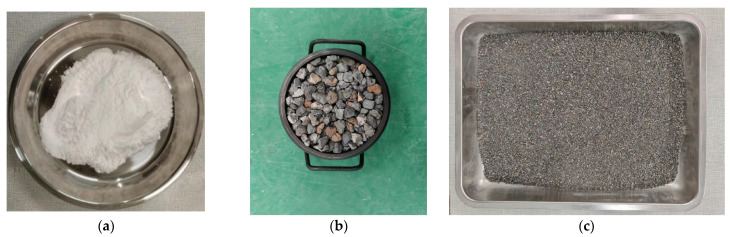
Physical pictures of (**a**) HGM; (**b**) SC; and (**c**) SPS.

**Figure 3:**
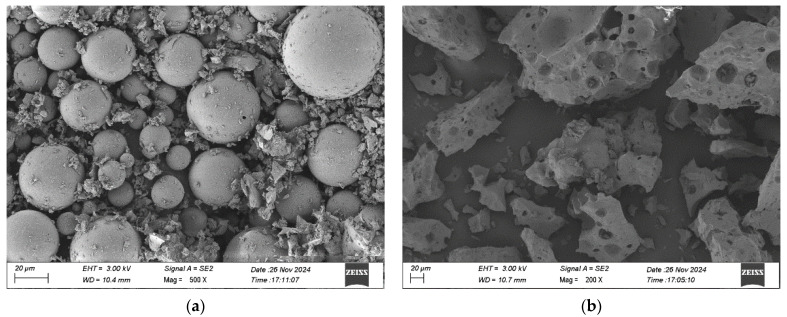
Microstructure of SEM: (**a**) HGM; (**b**) SPS.

Shale ceramsite sand (SPS) and shale ceramsite (SC) were supplied by Xinjiayuan Company (Gongyi, China), with SPS (0–5 mm) and SC (5–10 mm) selected as fine and coarse aggregates, respectively. The physical properties of LWA and HGM are summarized in Table 2. The bulk density, water absorption, cylinder compressive strength, and apparent density of LWA were determined in accordance with the Chinese standard JGJ/T 12-2019 [34]. The physical pictures of HGM, SC, and SPS are shown in Figure 2. In addition, scanning electron microscopy (SEM) images of SPS and HGM are shown in Figure 3.

The alkaline activator was prepared by dissolving Na_2_O·9SiO_2_ and K_2_CO_3_ (analytical grade, purity ≥ 99%, supplied by Tianjin Dingshengxin Chemical Company, Tianjin, China) in water at a mass ratio of 10:7.

### 2.2. Sample Preparation

The mixture proportions are detailed in Table 3. GGBFS was replaced by HGM at 3, 6, 9, and 12 wt%, designated as H3, H6, H9, and H12, respectively. SC was substituted with SPS at 35, 65, and 100 vol%, designated as S35, S65, and S100.

Prior to testing, SC was pre-soaked to saturated surface-dry condition to compensate for additional water absorption. The solid materials (GGBFS, SF, HGM) were mixed and dry-blended for 180 s. Subsequently, the prepared alkali-activator solution was gradually added, followed by mixing at low speed for 90 s and high speed for 30 s.

The homogeneous mixture was then cast into molds, sealed with plastic film, and demolded after 24 h of curing. Demolded specimens were cured in a standard conditioning chamber (20 ± 1 °C, 95 ± 5% RH) for 7 and 28 days.

### 2.3. Fluidity Test

The fluidity of the paste mixture was tested in accordance with the Chinese standard GB/T 50080-2016 [35]. A conical mold with a height of 300 mm, a top diameter of 100 mm, and a bottom diameter of 200 mm was utilized. The experimental procedure was conducted as follows: (i) The mixture was filled into the mold in three layers, with each layer compacted 25 times using a tamping rod from the periphery to the center. (ii) After filling and compacting the final layer, excess mixture was removed, and the surface was leveled flush with the mold rim. (iii) The conical mold was lifted vertically upward in a steady motion. Once the mixture ceased flowing, the maximum spread diameter and the perpendicular diameter were measured, and the average value was calculated.

### 2.4. Dry Density Test

The dry density of AAS-LWHSC was determined using the crushed specimen drying method in accordance with the Chinese standard JGJ/T 12-2019 [34]. The testing procedure was conducted as follows: (i) The surface moisture of the specimens was wiped off, and their weights were recorded. (ii) The length, width, and height of the specimens were measured using Vernier calipers. The average dimensions were calculated to determine the specimen volume, and the apparent density of the concrete in the natural state was computed based on the mean volume. (iii) The fractured specimens were homogenized, and 1 kg of fragments (20–30 mm in size) was selected. These fragments were dried to constant weight at 105 °C and subsequently weighed. (iv) The dry density of the concrete was calculated.

### 2.5. Mechanical and Physical Properties Test

The compressive strength, splitting tensile strength, and axial compressive strength of the specimens were tested using an electro-hydraulic servo press in accordance with the Chinese standard GB/T 50081-2019 [36]. Cubic specimens measuring 100 mm × 100 mm × 100 mm were employed for compressive strength testing, while 150 mm × 150 mm × 150 mm cubic specimens were utilized for splitting tensile strength evaluation. Prismatic specimens of 150 mm × 150 mm × 300 mm were designated for axial compressive strength assessment. The loading rates were set at 0.9 MPa/s, 0.09 MPa/s, and 0.1 mm/min for the three tests, correspondingly.

### 2.6. Microstructure Analysis

Microstructural analysis was conducted using a field-emission scanning electron microscope (SEM, Zeiss Sigma 500, ZEISS, Oberkochen, Germany). Small fragments were collected from the central region of the specimens after mechanical testing for observation. Prior to analysis, the samples were dried in an oven at 60 °C and subsequently sputter-coated with a gold layer to enhance surface conductivity.

## 3. Results and Discussion

### 3.1. Flowability

**Figure 4:**
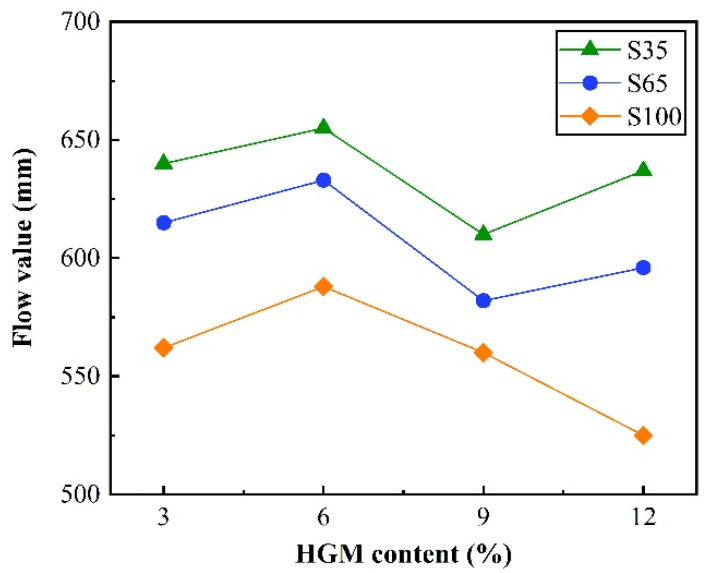
Flow value of the AAS-LWHSC.

Fluidity is recognized as a critical indicator of concrete workability. As illustrated in Figure 4, twelve groups of slump flow tests were conducted for AAS-LWHSC, with results ranging from 525 to 655 mm, demonstrating favorable fluidity.

Despite the low water-to-binder ratio (0.23) adopted in the test, adequate consistency was maintained in the mixture after HGM replacement of GGBFS, with no segregation observed between aggregates and paste. A consistent fluidity trend—initial increase, subsequent decrease, and final increase—was observed with rising HGM content for both shale ceramsite sand replacement rates (35% and 65%). However, at a 100% shale ceramsite sand replacement rate, fluidity exhibited an initial increase followed by a decline, ultimately reaching 525 mm. These discrepancies were attributed to the high water demand of HGM [37]: under a constant water-to-binder ratio, higher HGM incorporation was correlated with reduced fluidity. Although the spherical morphology and smooth surface of HGM were expected to enhance fluidity through a “ball-bearing” effect, this benefit was limited to specimens with shale ceramsite sand replacement rates below 65%. This limitation arose because pre-wetted shale ceramsite could release moisture to partially offset HGM’s water demand, whereas excessive replacement led to insufficient water compensation.

### 3.2. Dry Density

**Figure 5:**
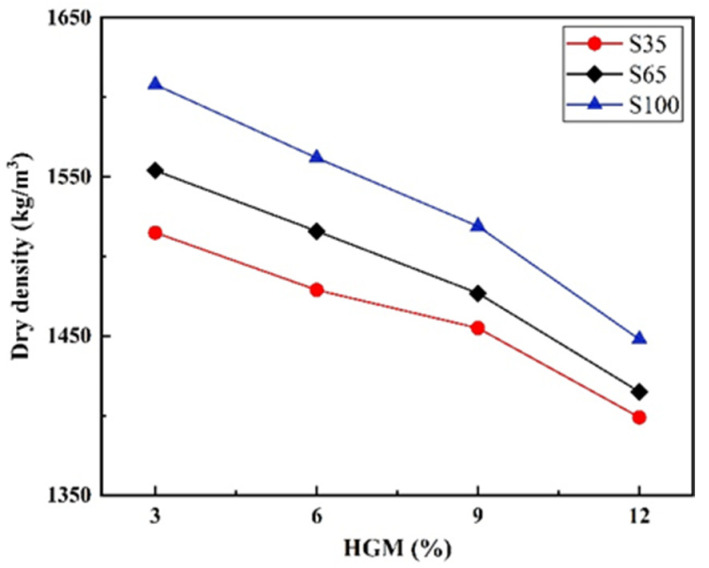
Dry density of the AAS-LWHSC.

Density is regarded as a critical parameter for evaluating LWHSC. As illustrated in Figure 5, dry density measurements were conducted across twelve groups of AAS-LWHSC specimens, yielding values ranging from 1399 to 1608 kg/m^3^, which conform to the requirements for LWHSC. The dry density was observed to decrease with increasing HGM dosage and increase with higher shale ceramsite sand replacement rates. For S35, S65, and S100 mixtures incorporating 12% HGM, reductions of 7.7%, 8.9%, and 10% in dry density were achieved compared to specimens with 3% HGM. The apparent density of concrete was significantly reduced by HGM, which is attributed to its inherently low apparent density. By replacing higher-density GGBFS with HGM, the overall weight of the concrete was effectively controlled. Furthermore, the synergistic combination of HGM and LWA was demonstrated to facilitate the production of AAS-LWHSC with further reduced dry density.

### 3.3. Compressive Strength

**Figure 6:**
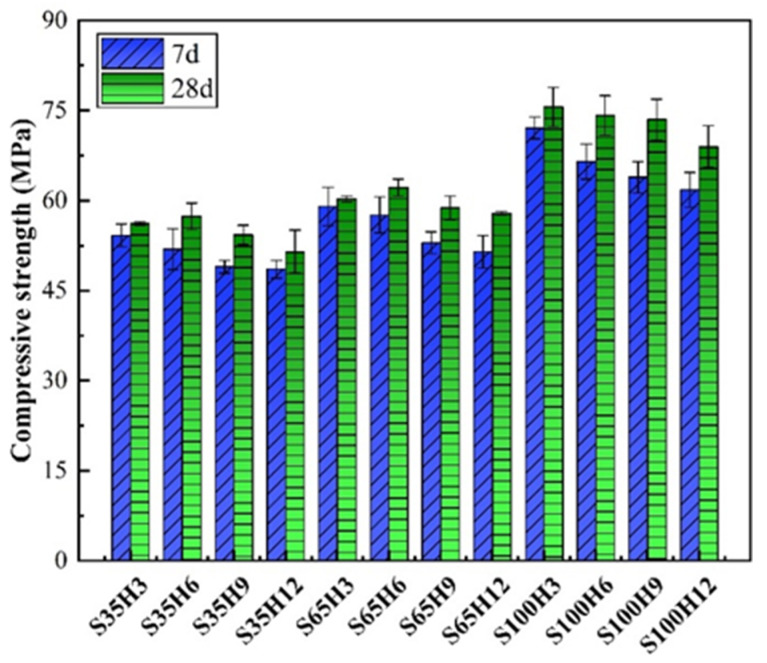
Compressive strength of the AAS-LWHSC.

Compressive strength testing of AAS-LWHSC was conducted across twenty-four groups. As illustrated in Figure 6, the 7-day and 28-day compressive strength results yielded ranges of 48.6–72.1 MPa and 51.5–75.6 MPa, respectively. The results indicated that the compressive strength of AAS-LWHSC was reduced with increasing HGM content, with the highest strength (75.6 MPa) observed in the S100H3 group. This reduction was primarily attributed to the hollow structure of HGM, which increased the internal porosity of the concrete and negatively affected compressive performance. However, the decline in compressive strength was relatively moderate, suggesting that high strength could still be maintained despite HGM replacing GGBFS.

When HGM dosage was increased from 3% to 6%, slight improvements in 28-day compressive strength were observed in the S35H6 and S65H6 groups, contrasting with the decreasing trend at 7 days. This phenomenon implies that the pozzolanic reactions of HGM may have been activated over prolonged curing periods, contributing to strength enhancement. Conversely, no strength improvement was detected in the S100H6 group compared to S100H3 at 28 days, indicating that the pozzolanic reactivity of HGM was constrained by insufficient water availability. The absence of the water-releasing effect from shale ceramsite in fully replaced S100 groups likely limited hydration completeness.

Furthermore, the significantly higher compressive strengths of S100 groups compared to S35 and S65 groups demonstrated that fine-grained LWAs could mitigate void defects caused by coarse aggregates, thereby optimizing the mechanical integrity of AAS-LWHSC.

### 3.4. Splitting Tensile Strength

**Figure 7:**
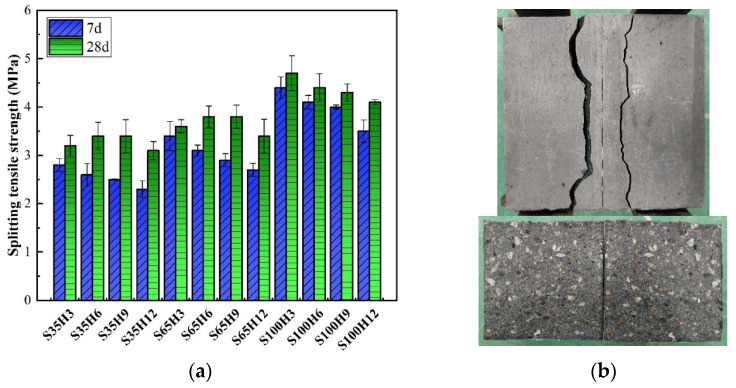
Splitting tensile test of the AAS-LWHSC: (**a**) splitting tensile strength; (**b**) failure mode of the AAS-LWHSC.

The splitting tensile strength of AAS-LWHSC was tested across twenty-four groups. As illustrated in Figure 7a, the 7-day and 28-day splitting tensile strength results yielded ranges of 2.3–4.4 MPa and 3.1–4.7 MPa, respectively. The 7-day splitting tensile strength was observed to decrease with increasing HGM content, aligning with the compressive strength trend. However, the 28-day splitting tensile strength was categorized into two distinct trends based on shale ceramsite sand replacement rates: an initial increase followed by a decrease in the S35 and S65 groups and a continuous decline in the S100 group.

This divergence was attributed to the pozzolanic reactivity of HGM. With extended curing, additional C-S-H gels were formed on the HGM shell surfaces at 28 days, refining the interfacial transition zone (ITZ) between HGM and the cementitious matrix. This enhanced ITZ enabled better distribution of localized stresses applied to HGM. Furthermore, the high energy absorption characteristics of HGM, derived from its spherical geometry and superior mechanical strength, were demonstrated to improve the deformation capacity and flexural resistance [38].

In contrast, no energy absorption benefits were observed in the S100 group. This was due to the dense packing of shale ceramsite sand as the sole aggregate, which restricted matrix deformation under load. Consequently, stress concentrations at the HGM-matrix interface caused premature crushing of HGM spheres, leading to reduced splitting tensile strength.

Failure modes and crack patterns observed from splitting tensile testing are depicted in Figure 7b. The variation in HGM dosage and SPS substitution rate did not alter the failure mode of the specimens, with all exhibiting brittle failure. The loaded surface of the AAS-LWHSC specimens was characterized by three primary cracks. The central crack symmetrically divided the specimen into two fragments. During failure, concrete in regions adjacent to the central crack was unable to withstand loads transferred from the central zone, resulting in secondary crack formation on both sides. Additionally, examination of post-failure cross-sections revealed clean fracture surfaces with regular geometry, while both fractured segments displayed relatively smooth internal surfaces devoid of irregular protrusions or depressions.

These observations further confirmed the predominance of brittle failure. Internally, SPS and SC were cleanly separated by propagating cracks during fracture, with no evidence of aggregate pull-out or ductile deformation.

### 3.5. Analysis of Damage Mode and Cracks

**Figure 8:**
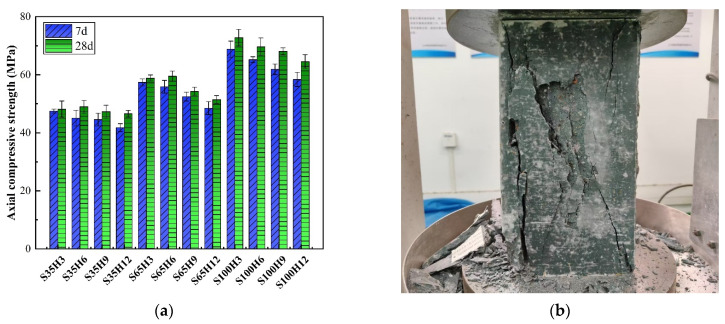
(**a**) Axial compressive strength and (**b**) failure mode of the AAS-LWHSC.

The axial compressive strength of AAS-LWHSC was tested across twenty-four groups. As illustrated in Figure 8a, the 7-day and 28-day strength ranges were measured as 41.8–68.8 MPa and 46.6–72.8 MPa, respectively. The failure modes and crack patterns observed from axial compressive testing are depicted in Figure 8b. AAS-LWHSC exhibited a brittle failure mode, characterized by no significant deformation, as observed during loading. However, fragment ejection and surface spalling were detected upon approaching peak load. Cracks manifested abruptly on both frontal and lateral surfaces, propagating rapidly.

The failure cracks were composed of vertical and diagonal cracks on the specimen surface. Diagonal cracks extended toward the specimen core, while vertical cracks penetrated the entire cross-section. The formation of vertical cracks was attributed to the limited interfacial bonding between HGM and the surrounding matrix, caused by the spherical morphology and smooth surface of HGM, which failed to restrict crack development [39].

### 3.6. Microstructure

**Figure 9:**
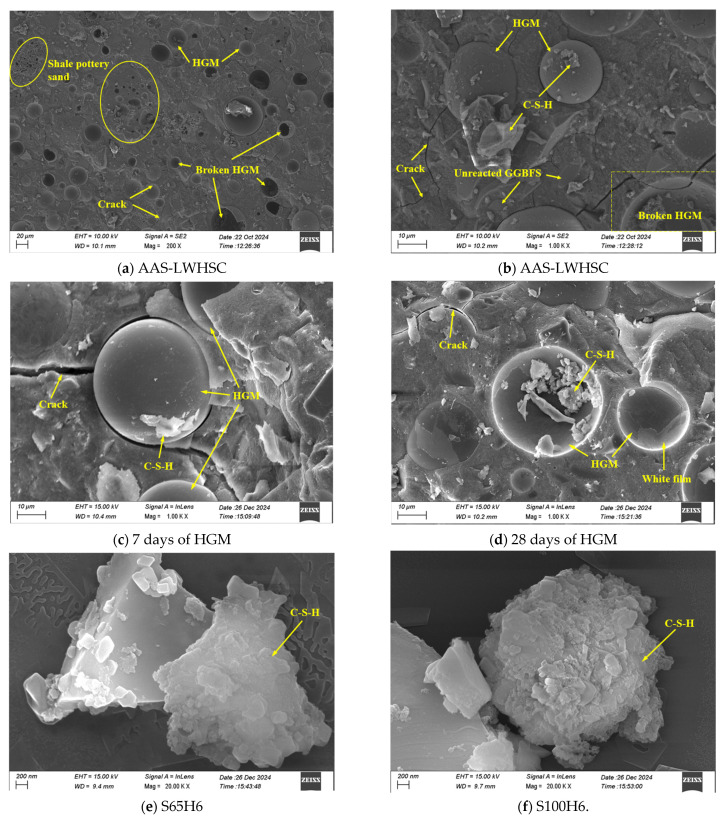
Microscopic electron microscope SEM: (**a**,**b**) AAS-LWHSC; (**c**) 7 days of HGM; (**d**) 28 days of HGM; (**e**) S65H6; (**f**) S100H6.

Figure 9a illustrates the microstructural morphology of AAS-LWHSC, where HGM and SPS were uniformly distributed within the matrix. SPS was tightly bonded to the matrix, exhibiting a smooth interfacial transition zone (ITZ). HGM was perfectly embedded in the matrix with strong interfacial adhesion, and no debonding phenomena were detected. The dark cavities observed were identified as residual HGM fragments left after mechanical failure. Figure 9b reveals that not all HGM particles were fractured under loading, indicating that the high-strength shell of HGM resisted stress transfer from the surrounding matrix, which partially explains why mechanical properties were retained despite HGM incorporation.

Figure 9c,d present the surface morphology of HGM spheres at 7 and 28 days of curing, respectively. At 7 days, partial gel attachment was observed on HGM surfaces, which remained relatively smooth, suggesting limited pozzolanic reactivity at early stages and the corresponding reductions in mechanical performance. By 28 days, the gel coverage was significantly increased, and a white film formed on HGM surfaces due to pozzolanic reactions, enhancing surface roughness and improving interfacial bonding with the matrix.

Figure 9e,f compare the morphologies of C-S-H gels on HGM surfaces in the S65H6 and S100H6 groups after 28 days of curing. In S65H6, the C-S-H gels exhibited smoother surfaces with fewer wrinkles, indicative of advanced hydration and higher reaction completeness. Conversely, the C-S-H gels in S100H6 displayed rougher, multilayered structures resembling aggregated small gel clusters, reflecting inferior hydration. This disparity was attributed to the absence of water-releasing effects from shale ceramsite in S100H6, which restricted additional moisture availability for further hydration product development. Enhanced hydration of C-S-H gels on HGM surfaces strengthened interfacial bonding and stress transfer efficiency, explaining the mechanical performance improvements in S35 and S65 groups with 6% HGM.

## 4. Discussion

The spherical structure of HGM exhibits a dual nature. At SPS replacement ratios < 65%, incorporation of 3% HGM is observed to enhance initial flowability via the “ball-bearing effect”, whereby interparticle friction is reduced. Furthermore, at 28-day curing ages, the perfect sphericity and high-strength shell are demonstrated to increase energy absorption capacity, enabling effective stress distribution and improved splitting tensile strength.

However, the hollow spherical structure inherently increases concrete porosity, resulting in mechanical properties being compromised. Additionally, both the ball-bearing effect and pozzolanic reactivity of HGM are critically influenced by the water-releasing mechanism of pre-wetted SPS. Water released from SPS compensates for absorption by HGM, thereby sustaining the ball-bearing effect while simultaneously promoting pozzolanic reactions.

This synergistic mechanism facilitates enhanced formation of C-S-H gels on sphere surfaces and enables a more thorough hydration, ultimately contributing to strength development at later curing stages.

## 5. Conclusions

Dual-phase optimization of HGM and LWA: Through the synergistic interaction at 6% HGM dosage and 35–65% shale ceramsite sand replacement ratio, flowability is enhanced (via the “ball-bearing effect”), while the 28-day strength is improved (through controlled pozzolanic reactivity).

Density reduction while maintaining strength: The dry density of AAS-LWHSC is significantly reduced to 1608 kg/m^3^ through hierarchical replacement of GGBFS by HGM and rational LWA combination, with 75.6 MPa compressive strength retained.

Criticality of water management mechanism: The water-releasing effect of shale ceramsite is identified as essential for activating HGM’s pozzolanic reactivity. During curing, adequate water availability is demonstrated to enable more complete development of pozzolanic reactions—a previously overlooked factor in alkali-activated slag lightweight high-strength concrete systems.

Dual functionality of HGM: (i) Energy absorption through spherical integrity under stress is achieved at 35% and 65% shale ceramsite sand replacement ratios. (ii) The interfacial transition zone (ITZ) is refined via C-S-H growth on sphere surfaces, strengthening matrix bonding and offsetting strength loss induced by porosity.

## Figures and Tables

**Table 1:** Chemical composition of GGBFS, SF, and HGM (% by weight).

	CaO	SiO_2_	Al_2_O_3_	MgO	K_2_O	Na_2_O	Fe_2_O_3_	SO_3_
GGBFS	39.29	33.06	15.04	9.96		0.43	0.32	1.9
SF	0.67	93.7	0.3	0.69	1.17	0.79	2.38	0.3
HGM	8.04	80.1	0.75	0.18	0.03	10.61	0.05	0.24

**Table 2:** Physical properties of HGM, SPS, and SC.

	Bulk Density (kg/m^3^)	Density (kg/m^3^)	Tube Crushing Strength (MPa)	1 h Water Absorption (%)
HGM	0.39	0.60	82	
SPS	827	1545		
SC	712	1307	6	4.2

**Table 3:** Mix proportions (by weight).

Group	GGBFS (kg/m^3^)	SF (kg/m^3^)	HGM (kg/m^3^)	SPS (kg/m^3^)	SC (kg/m^3^)	H_2_O (kg/m^3^)	Na_2_O·9SiO_2_ (kg/m^3^)	K_2_CO_3_ (kg/m^3^)
S35H3	636.09	165.22	24.78	216.30	352.89	190.00	83.44	58.57
S35H6	611.30	165.22	49.57	200.85	326.75	190.00	83.44	58.57
S35H9	586.52	165.22	74.35	185.40	300.61	190.00	83.44	58.57
S35H12	561.74	165.22	99.13	169.95	261.40	190.00	83.44	58.57
S65H3	636.09	165.22	24.78	417.15	182.98	190.00	83.44	58.57
S65H6	611.30	165.22	49.57	386.25	169.91	190.00	83.44	58.57
S65H9	586.52	165.22	74.35	355.35	156.84	190.00	83.44	58.57
S65H12	561.74	165.22	99.13	309.00	143.77	190.00	83.44	58.57
S100H3	636.09	165.22	24.78	633.45	0	190.00	83.44	58.57
S100H6	611.30	165.22	49.57	587.10	0	190.00	83.44	58.57
S100H9	586.52	165.22	74.35	540.75	0	190.00	83.44	58.57
S100H12	561.74	165.22	99.13	478.95	0	190.00	83.44	58.57

## Data Availability

The original contributions presented in this study are included in the article. Further inquiries can be directed to the corresponding author.
